# Association Between Serum Lipid Levels, Resilience, and Self-Esteem in Japanese Adolescents: Results From A-CHILD Study

**DOI:** 10.3389/fpsyg.2020.587164

**Published:** 2021-01-12

**Authors:** Satomi Doi, Aya Isumi, Takeo Fujiwara

**Affiliations:** ^1^Department of Global Health Promotion, Tokyo Medical and Dental University, Tokyo, Japan; ^2^Japan Society for the Promotion of Science, Tokyo, Japan

**Keywords:** adolescent, serum lipid levels, cholesterol, mental health, Japan

## Abstract

Previous studies have found that serum lipid levels independently associate with mental health problems in adulthood. However, little is known about the association between serum lipid levels and positive aspects of mental health such as resilience and self-esteem, which develop in adolescence. The aim of this study is to examine the association between serum lipid levels and resilience and self-esteem in Japanese adolescents. Data were pooled data from the Adachi Child Health Impact of Living Difficulty (A-CHILD) study in 2016 and 2018, a school-based, cross-sectional study in Adachi City, Tokyo, Japan (*N* = 1,056, aged 13–14 years). Resilience of the child was assessed by caregivers, and self-esteem was self-identified *via* questionnaires. Serum lipid levels [total cholesterol, low-density lipoproteins (LDL), and high-density lipoproteins (HDL)] were assessed in school health checkup, in addition to height and weight measurements. Multiple linear regression was applied to investigate the association between standardized serum lipid levels and resilience and self-esteem. LDL showed inverse association with resilience [β = −1.26, 95% confidence interval (CI) = −2.39 to −0.14] after adjusting for child’s BMI, month of birth, sex, absence of parent, household income, caregiver’s mental health, and lifestyle (e.g., habits of eating, physical activity, and sleep). We also found an inverse association of total cholesterol and higher LDL cholesterol with self-esteem (β = −0.58, 95% CI = −0.99 to −0.18; β = −0.42, 95% CI = −0.83 to −0.01, respectively). HDL cholesterol was not associated with resilience and self-esteem. Among Japanese adolescent, total and LDL cholesterol may be biomarkers of resilience and self-esteem.

## Introduction

Mental health problems such as depression and anxiety are a common and major public health issue in adolescents (Werner-Seidler et al., 2017). According to a large longitudinal study conducted in 11 counties in western North Carolina, United States, it was estimated that the overall prevalence of depression was 2.8% for children under 13 years old and 5.6% for young people 13–18 years of age (Costello et al., 2003). Furthermore, depression and anxiety appear by the age of 18 years in up to 20% of young people in the United States (Lewinsohn et al., 1998; de Girolamo et al., 2012). The onset of depression and anxiety during adolescence leads to dire clinical outcomes throughout the course of their life. In addition to mental disorders such as depression and anxiety disorders (Kessler et al., 2001), dependent disorders (Weissman et al., 1999), and suicidality (Weissman et al., 1999), there are physical ailments such as cardiovascular disease (Janszky et al., 2010). Therefore, it is important to prevent mental health problems in adolescence.

Resilience and self-esteem are critical tools in tackling stress, a catalyst for mental health problems. Resilience is defined as “a dynamic process wherein individuals display positive adaptation despite experiences of significant adversity or trauma” (Rutter, 1999; Lutha and Cicchetti, 2000). There are programs to enhance the development of resilience. Many have proven to be effective in preventing mental health problems including depression (Brunwasser et al., 2009) and anxiety (Gillham et al., 2006) and academic failure (Luthar and Ansary, 2005) in adolescents. An important attribute of resilient individuals is self-esteem (Masten and Coatsworth, 1998), which is defined as “an individual’s subjective evaluation of her or his worth as a person” (Donnellan et al., 2011). Higher self-esteem is negatively associated with mental health problems including depression (Moksnes et al., 2010), anxiety (Moksnes et al., 2010), risky behaviors (Veselska et al., 2009), and suicidality (Sharaf et al., 2009).

Serum lipid levels including total cholesterol, high-density lipoprotein (HDL), and low-density lipoprotein (LDL) may serve as biomarkers to monitor mental health problems. Previous studies have found that total and LDL cholesterol independently correlate with depression (Partonen et al., 1999; Wysokinski et al., 2015; Parekh et al., 2017; Wagner et al., 2019; Wei et al., 2020), schizophrenia (Wysokinski et al., 2015), or suicidality (Partonen et al., 1999) in adults. Unfortunately, little is known about the association between serum lipid levels and positive aspects of mental health including resilience and self-esteem.

The International Survey of Youth Attitude in 2013 showed that Japanese adolescents displayed larger variance on self-esteem in comparison with their counterparts in South Korea, the United States, and European countries (The Cabinet Office Government of Japan, 2014). Among Japanese aged 13–29 years, 45.8% responded to the item on self-esteem (i.e., “I am satisfied with myself”) with “I agree” or “I kind of agree,” as opposed to 71.5% in South Korea, 86.0% in the United States, 83.1% in the United Kingdom, 80.9% in Germany, 82.7% in France, and 74.4% in Sweden (The Cabinet Office Government of Japan, 2014). In view of this finding, we postulate that the Japanese population is suitable for the investigation of the association between serum lipid levels and positive aspects of mental health.

## Materials and Methods

### Participants

**FIGURE 1:**
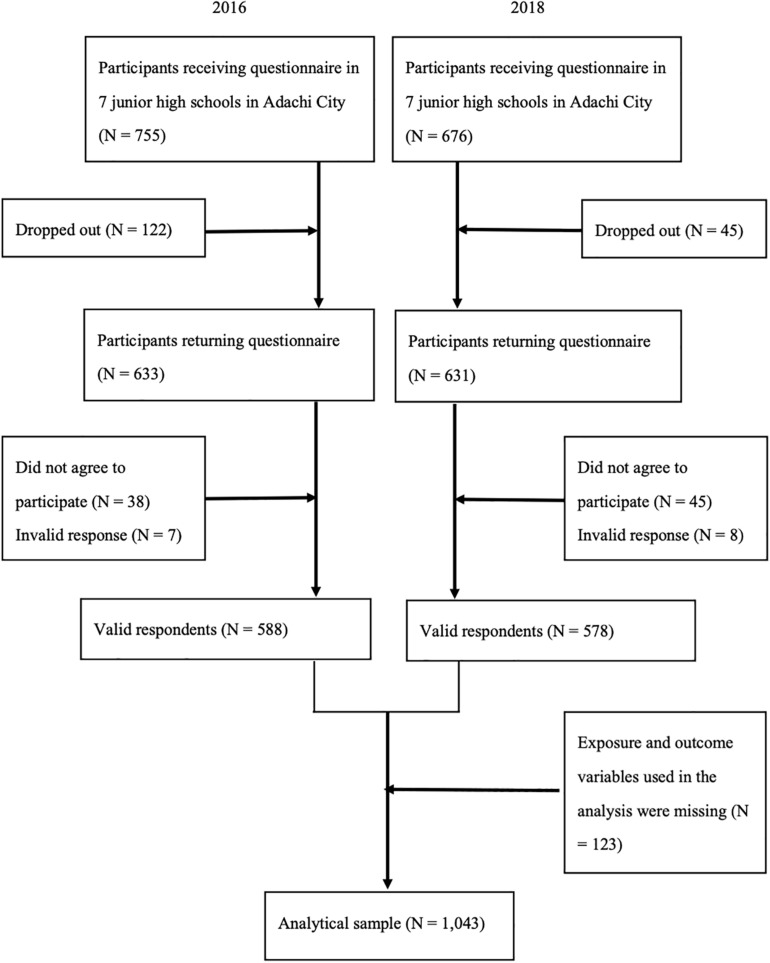
Participant flow chart.

Data were pooled data from the Adachi Child Health Impact of Living Difficulty (A-CHILD) study, a school-based longitudinal study examining the living environment and health of elementary school and junior high school students and their parents in Adachi City, Tokyo, Japan. The details of the cohort profiles have been reported somewhere (Ochi et al., 2020). We used data of children aged 13–14 years (i.e., eighth grade) in seven representative junior high schools in Adachi City, collected in 2016 and 2018. Cross-sectional data were pooled to maximize sample size. Adolescents brought anonymous self-reported questionnaires with unique ID’s home to their caregivers in October. The questionnaires were distributed to 755 adolescents in 2016 and to 676 adolescents in 2018. The adolescents who returned both adolescent’s and their caregiver’s questionnaires with valid responses (i.e., with at least one response, obtained informed consent, and being able to link with school health checkup) were 588 (response rate: 77.9%) in 2016 and 578 (response rate: 85.5%) in 2018. Among the valid responses, we excluded the participants who missed exposure and outcome variables in this study (i.e., total cholesterol, HDL, LDL, self-esteem, and resilience). Finally, our analytical sample comprised 1,043 adolescents (Figure 1).

### Measurements

Serum lipid levels including total, LDL, and HDL cholesterol were assessed during school health checkups conducted in June 2016 and 2018. We took a venipuncture blood sample from the arm and measured cholesterol level at the laboratory. The students were not required to fast prior to the blood test. Total and HDL cholesterol were measured by enzymatic method (Nordestgaard, 2017). LDL cholesterol was assessed using a direct method in the pediatric population (Harris et al., 1996).

Resilience of the child was assessed by caregivers *via* questionnaires, using the Children’s Resilient Coping Scale (CRCS) which has high internal consistency (Doi et al., 2018). The CRCS consists of eight items (e.g., “speaks positively about their future,” “able to get ready for school, study, and do his/her chores without directions,” or “able to give up on things they want or do things that they do not like to do for better future outcomes”) and is rated using a scale of 0 (never) to 4 (very frequently). Total score of the CRCS were converted into 0 to 100. A higher total score denoted a higher level of child resilience. The Cronbach’s alpha for the scale was 0.87 in this study.

Self-esteem of the child was assessed by the subjects themselves *via* questionnaires, using the Japanese version of the Children’s Perceived Competence Scale (Sakurai, 1992). This scale is composed of 10 items (e.g., “are you satisfied with the way you are now?” or “do you think you have few good points?”) and is rated using a scale of 0 (no) to 3 (yes). A higher score denoted a higher level of self-esteem. The Cronbach’s alpha for the scale was 0.88 in this study.

Participants’ basic characteristics and covariates were assessed by both adolescents and their caregivers. That is, child sex (“male” or “female”), frequency of having breakfast (“always,” “sometimes,” “not very often,” or “never”), frequency of physical activity (“not at all,” “1–2 times a week,” “3–4 times a week,” “5–6 times a week,” or “7 or more times a week”), bedtime difference between school night and holiday (“<2” or “2 h+”), wake-up time difference between school day and holiday (“<2” or “2 h+”) were assessed by adolescent *via* questionnaire. Members living together, birth month of child, maternal age, paternal age, maternal weight and height, maternal history of diabetes, maternal history of cardiovascular disease, paternal history of diabetes, paternal history of cardiovascular disease, respondent’s mental health assessed using the Japanese version of the Kessler 6 (K6), frequency of eating vegetable in child (“almost both breakfast and dinner,” “everyday, either breakfast or dinner,” “2–3 meals per week,” or “less than 1 meal per week”), and annual household income were assessed by caregiver *via* questionnaire. Furthermore, child weight and height data were collected in school health checkups and calculated z-score of BMI based on WHO Child Growth Standards specific to age and sex (de Onis et al., 2007).

### Ethics Statement

This study was approved by the Ethics Committee of the Tokyo Medical and Dental University (M2016-284-05).

### Statistical Analysis

In the analyses, each serum lipid level (i.e., total, LDL, and HDL cholesterol) was standardized for comparability, that is, divided by one standardized deviation (SD). The missing data in covariates were treated as a dummy variable. First, we performed residual analysis to check regression assumptions. According to the visual examination of Q–Q plots and residual plot, the assumptions for regression analysis were met. Second, the Spearman’s rank correlation was performed to identify the covariates which were added into the analysis. In addition to the results of the Spearman’s rank correlation, covariates were selected based on biological plausibility and previous studies which examined the association between serum lipid level and mental health problems (Shin et al., 2008; Kong et al., 2011; Fang et al., 2013; Park et al., 2020). Third, linear regression analysis was conducted to investigate the crude association of serum lipid levels with resilience and self-esteem. Fourth, we performed adjusted models for each serum lipid level and each outcome, adjusted for covariates which showed *r* > 0.10 in the Spearman’s rank correlation or might need to be added according to previous studies. Variance inflation factor (VIF) values were calculated to assess the multicollinearity for regression models. Furthermore, we performed adjusted models using the complete data for the sensitivity analysis. All analyses were conducted using STATA version 15.0 SE.

## Results

Table 1 shows the distribution of characteristics among all participants. About 90% of caregivers who responded to the questionnaire were mothers. The means and SDs of total, LDL, and HDL cholesterol were 167.02 (SD = 26.79), 91.25 (SD = 23.18), and 62.51 (SD = 11.98), respectively. Regarding outcome variables, the means and SDs of resilience and self-esteem were 67.57 (SD = 18.28) and 14.24 (SD = 6.55), respectively. Over 30% of participants were out of the normal BMI range. Approximately 15% of participants did not have breakfast every day, 2% ate vegetable less than 1 meal/week (caregiver-reported), 30% did not participate in any physical activities, 3% went to bed at irregular timings, and 40% woke up at irregular timings. A total of 20% of participants did not live with their mother or/and father, 15% of the mothers were less than 40 years old, 10% of the fathers were less than 40 years old, 20% of the mothers were out of the normal BMI range, 1% of the mothers had the history of diabetes, 2% of mothers had history of cardiovascular disease, 3% of fathers had the history of diabetes, 3% of fathers had the history of cardiovascular disease, 35% of participants reported psychological distress, and 10% had low household income (<JPY3,000,000).

**TABLE 1:** Characteristics of sample (*N* = 1,043).

	*N* (Mean)	% (SD)
**Health check-up**		
Total cholesterol	167.02	26.79
LDL cholesterol	91.25	23.18
HDL cholesterol	62.51	11.98
Child’s BMI		
≤1 SD	185	17.7
−1 SD ≤ 1 SD	693	66.4
1 SD+	143	13.7
Missing	22	2.1
**Assessed by children**		
Sex of child		
Male	493	47.3
Female	549	52.6
Missing	1	0.1
Frequency of having breakfast		
Always	858	82.3
Sometimes	115	11.0
Not very often/Never	61	5.8
Missing	9	0.9
Frequency of physical activity		
3 times or more per week	507	48.6
1–2 times per week	208	19.9
Almost no or not at all	322	30.9
Missing	6	0.6
Irregular bedtime (bedtime difference between school night and holiday)		
<2 h	1,005	96.4
2 h+	27	2.6
Missing	11	1.0
Irregular wake-up time (time difference between school and holiday wake-up)		
<2 h	577	55.3
2 h +	455	43.6
Missing	11	1.0
**Assessed by caregivers**		
Respondent		
Mother	932	89.4
Father	86	8.2
Grandmother	8	0.8
Grandfather	2	0.2
Relative	3	0.3
Other	4	0.4
Missing	8	0.8
Absence of mother and/or father		
No	840	80.5
Yes	203	19.5
Maternal age		
<35 years	38	3.6
35 ≤ 40 years	126	12.1
40 ≤ 45 years	389	37.3
45 years+	446	42.8
Missing	44	4.2
Paternal age		
<35 years	19	1.8
35 ≤ 40 years	80	7.7
40 ≤ 45 years	267	25.6
45 years+	498	47.7
Missing	179	17.2
Maternal BMI		
<18.5	104	10.0
18.5 ≤ 25.0	716	68.7
25.0 ≤ 30.0	100	9.6
30.0+	20	1.9
Missing	103	9.9
Maternal history of diabetes		
Yes	13	1.2
No	1,030	98.8
Maternal history of cardiovascular disease		
Yes	19	1.8
No	1,024	98.2
Paternal history of diabetes		
Yes	32	3.1
No	1,011	96.9
Paternal history of cardiovascular disease		
Yes	27	2.6
No	1,016	97.4
Respondent’s mental health (K6)		
<5	657	63.0
5 ≤ 13	296	28.4
13+	69	6.6
Missing	21	2.0
Frequency of eating vegetable in child		
Almost both breakfast and dinner	405	38.8
Everyday, either breakfast or dinner/2–3 meals per week	607	58.2
Less than 1 meal per week	21	2.0
Missing	10	1.0
Household income		
<3,000,000	124	11.9
3,000,000 ≤ 6,000,000	337	32.3
6,000,000–10,000,000	343	32.9
10,000,000+	90	8.6
Unknown	149	14.3

Table 2 shows the results of the Spearman’s rank correlation to examine the association between covariates and serum lipid level, resilience, and self-esteem. Child’s BMI, child’s sex, frequency of having breakfast, and frequency of physical activity were significantly associated with both exposures (i.e., any of total cholesterol, LDL cholesterol, and HDL cholesterol) and outcomes (i.e., any of resilience and self-esteem) (*r* > 0.10). According to these results and previous studies (Shin et al., 2008; Kong et al., 2011; Fang et al., 2013; Park et al., 2020), child’s BMI, child’s month of birth, child’s sex, absence of mother or/and father, household income, respondent’s mental health, frequency of eating vegetable in child, frequency of having breakfast, frequency of physical activity, regularity of bedtime, and regularity of wake-up time were added into the multiple linear regression analyses as possible confounders.

**TABLE 2:** Results of Spearman’s rank correlation.

	Total cholesterol	LDL cholesterol	HDL cholesterol	Resilience	Self-esteem
Child’s BMI	–0.01	0.09**	−0.19**	−0.07*	−0.12**
Child’s month of birth	–0.03	–0.002	−0.06*	0.02	0.05
Sex of child	0.30**	0.25**	0.18**	0.18**	−0.11**
Absence of mother or/and father	0.03	0.06	–0.06	–0.06	–0.04
Maternal age	–0.02	0.003	–0.05	–0.01	–0.01
Paternal age	0.001	0.01	–0.04	–0.03	–0.05
Household income	0.03	–0.01	0.04	0.08*	0.04
Respondent’s mental health (K6)	0.03	0.02	0.003	−0.16**	–0.05
Frequency of eating vegetable in child	0.02	0.05	–0.02	−0.16**	−0.11**
Frequency of having breakfast	0.12**	0.17**	–0.05	−0.10**	−0.14**
Frequency of physical activity	0.08**	0.17**	−0.15**	–0.05	−0.18**
Regularity of bedtime	0.05	0.03	0.04	–0.05	–0.05
Regularity of wake-up time	0.10**	0.12**	–0.06	−0.09**	−0.08**
Maternal BMI	0.03	0.08*	−0.09**	–0.05	−0.12**
Maternal history of diabetes	0.001	0.01	–0.04	–0.06	−0.06*
Maternal history of cardiovascular disease	0.04	0.04	0.02	–0.04	–0.03
Paternal history of diabetes	0.01	0.001	–0.02	–0.06	0.01
Paternal history of cardiovascular disease	0.04	0.05	0.02	–0.06	0.04

*^∗^*p* < 0.05; ^∗∗^*p* < 0.01.*

Table 3 shows the results of the multiple linear regression analyses to examine the association between serum lipid levels and resilience. In the crude model, LDL cholesterol was negatively associated with resilience (β = −1.30, 95% confidential interval (CI) = −2.41 to −0.19), and HDL cholesterol was positively associated with resilience (β = 2.15, 95% CI = 1.04 to 3.25). In the adjusted models, the coefficient of LDL cholesterol remained significant (β = −1.26, 95% CI = −2.39 to −0.14). In contrast, the coefficient of HDL cholesterol was not significant in the adjusted model (β = 0.68, 95%CI = −0.68 to 1.82).

**TABLE 3:** The associations between serum lipid level and resilience.

	Crude model	Total cholesterol, adjusted model	LDL cholesterol, adjusted model	HDL cholesterol, adjusted model
	β (95% CI)	β (95% CI)	β (95% CI)	β (95% CI)
Total cholesterol (1 SD)	−0.29 (−1.40 to 0.82)	−0.95 (−2.07 to 0.17)		
LDL cholesterol (1 SD)	−1.30 (−2.41 to −0.19)		−1.26 (−2.39 to −0.14)	
HDL cholesterol (1 SD)	2.15 (1.04 to 3.25)			0.68 (−0.46 to 1.82)

Table 4 shows the results of the multiple linear regression analyses to examine the association between serum lipid levels and self-esteem. In the crude model, total cholesterol and LDL cholesterol were negatively associated with self-esteem (total cholesterol: β = −0.83, 95% CI = −1.22 to −0.43; LDL cholesterol: β = −0.88, 95% CI = −1.28 to −0.49). In the adjusted models, both coefficients of total and LDL cholesterol remained significant (total cholesterol: β = −0.58, 95% CI = −0.99 to −0.18; LDL cholesterol: β = −0.42, 95% CI = −0.83 to −0.01). However, HDL cholesterol was not associated with self-esteem.

**TABLE 4:** The associations between serum lipid levels and self-esteem.

	Crude model	Total cholesterol, adjusted model	LDL cholesterol, adjusted model	HDL cholesterol, adjusted model
	β (95% CI)	β (95% CI)	β (95% CI)	β (95% CI)
Total cholesterol (1 SD)	−0.83 (−1.22 to −0.43)	−0.58 (−0.99 to −0.18)		
LDL cholesterol (1 SD)	−0.88 (−1.28 to −0.49)		−0.42 (−0.83 to −0.01)	
HDL cholesterol (1 SD)	−0.01 (−0.40 to 0.39)			−0.36 (−0.78 to 0.05)

The range of VIF values for each indicator in the regression models was less than five. Furthermore, using the complete data (*n* = 833), the analyses showed similar results (data not shown).

## Discussion

In the current study, we found an inverse association between LDL cholesterol and resilience. We also found an inverse association of total cholesterol and LDL cholesterol with self-esteem. These results remained significant after adjusting for child’s BMI, month of birth, sex, absence of parent, household income, caregiver’s mental health, and lifestyle (e.g., habits of eating, physical activity, and sleep), which were all associated with serum lipid levels (Kong et al., 2011; Hallstrom et al., 2013; Moschonis et al., 2013; Slopen et al., 2013; Oluwagbemigun et al., 2019; Ziaei et al., 2019), that is, total and LDL cholesterol independently associated with resilience and self-esteem.

These results indicated that total and LDL cholesterol serve as biomarkers of resilience and self-esteem among Japanese adolescents. Programs that promote positive aspects of mental health such as self-esteem have been developed for adolescents to prevent mental health problems. Usually, self-reported measurements of resilience and self-esteem are used to evaluate the efficacy of the prevention program (van Genugten et al., 2017). However, such evaluations have limitations, especially when used on adolescents. Adolescents may be less likely to express their mental health status with or without intention (Walker et al., 2017). In fact, early onset of mental health problems is associated with a longer duration of untreated illness (de Girolamo et al., 2012), which may be caused by a lack of accuracy of self-reported measurements. Thus, there is a need to evaluate potential biomarkers in addition to self-reported measurements to overcome this limitation. Our findings may support the feasibility of total and LDL cholesterol as biomarkers of resilience and self-esteem in adolescents.

Although we found the association of cholesterol with resilience and self-esteem, the mechanism of the association remains unclear. In the context of mental health problems such as depression, suicide, and aggression, the relationship between cholesterol and mental health problems is well-documented, but little is known about the mechanism. Pereira (2017) suggested that cholesterol may induce chemical changes and affect the likelihood of certain behavioral outcomes although it does not directly cause behaviors. Alternatively, previous studies indicated the association between serum lipid levels and depression-related inflammation (Maes et al., 1997; de Melo et al., 2017), with serum lipid levels being a biomarker of inflammation related to mental health. Additionally, recent studies have found the association between resilience and systematic inflammation such as anti-inflammatory interleukin-10 (IL-10) (Teche et al., 2017). Therefore, because poor resilience or low self-esteem can be considered prodrome of depression (Roberts, 2006), the association between serum lipid levels and self-esteem might be explained by the inflammation response pathway. Therefore, further study to elucidate the mechanism of the association between serum lipid levels and resilience or self-esteem, with a focus on inflammation, is warranted. In a further study, it is necessary to take into account the impact of genes which may determine resilience (Okbay et al., 2016; Feder et al., 2019; Stein et al., 2019).

The current study has several limitations. First, as this is a cross-sectional study, we cannot indicate the causal relationship of cholesterol with resilience and self-esteem. Previous studies have indicated that adolescents with higher self-esteem are more likely to engage in healthy behaviors including healthy eating (Martyn-Nemeth et al., 2009; Logi Kristjánsson et al., 2010). That is, adolescents with higher self-esteem might be less likely to eat food with high fat. Second, sampling bias might exist even though the response rate in this study was not low. Adolescents and caregivers who lived in poverty or had mental health problems might be less likely to respond to the questionnaire. Thus, our results might be underestimated by this selection bias. Third, as our results were derived from the data of Japanese adolescents aged 13–14 years living in a single community, the generalizability of our findings is precluded. Further studies in other settings are needed. Fourth, we only assessed total, LDL, and HLD cholesterol as biomarkers in this study. There are other possible biomarkers such as very-low-density lipoprotein (VLDL) which has shown the association with autism spectrum disorder (Usui et al., 2020). Further studies are needed to identify other biomarkers of mental health including resilience and self-esteem.

In conclusion, total and LDL cholesterol may be one biomarker of resilience and self-esteem among Japanese adolescents. Our findings may contribute to the evaluation of the efficacy of programs designed to promote resilience and self-esteem in adolescents.

## Data Availability Statement

The raw data supporting the conclusions of this article will be made available by the authors, without undue reservation.

## Ethics Statement

The studies involving human participants were reviewed and approved by Ethics Committee of the Tokyo Medical and Dental University (M2016-284-05). Written informed consent to participate in this study was provided by the participants’ legal guardian/next of kin.

## Author Contributions

TF designed the study. AI, SD, and TF managed administration of the study, including the ethical review process. SD analyzed data and drafted the manuscript. AI and TF provided critical comments on the manuscript related to intellectual content. All authors have read and approved the final manuscript.

## Conflict of Interest

The authors declare that the research was conducted in the absence of any commercial or financial relationships that could be construed as a potential conflict of interest.
